# MicroNIR/Chemometrics Assessement of Occupational Exposure to Hydroxyurea

**DOI:** 10.3389/fchem.2018.00228

**Published:** 2018-06-19

**Authors:** Roberta Risoluti, Stefano Materazzi

**Affiliations:** Department of Chemistry, Sapienza - University of Rome, Rome, Italy

**Keywords:** MicroNIR, chemometrics, hydroxyurea, occupational exposure, pharmaceutics

## Abstract

Portable Near Infrared spectroscopy (NIRs) coupled to chemometrics was investigated for the first time as a novel entirely on-site approach for occupational exposure monitoring in pharmaceutical field. Due to a significant increase in the number of patients receiving chemotherapy, the development of reliable, fast, and on-site analytical methods to assess the occupational exposure of workers in the manufacture of pharmaceutical products, has become more and more required. In this work, a fast, accurate, and sensitive detection of hydroxyurea, a cytotoxic antineoplastic agent commonly used in chemotherapy, was developed. Occupational exposure to antineoplastic agents was evaluated by collecting hydroxyurea on a membrane filter during routine drug manufacturing process. Spectra were acquired in the NIR region in reflectance mode by the means of a miniaturized NIR spectrometer coupled with chemometrics. This MicroNIR instrument is a very ultra-compact portable device with a particular geometry and optical resolution designed in such a manner that the reduction in size does not compromise the performances of the spectrometer. The developed method could detect up to 50 ng of hydroxyurea directly measured on the sampling filter membrane, irrespective of complexity and variability of the matrix; thus extending the applicability of miniaturized NIR instruments in pharmaceutical and biomedical analysis.

## Introduction

Hydroxyurea (HU) or hydroxycarbamide, is a non-alkylating hydroxylated urea analog mainly recognized as antineoplastic and antiviral agent (Spivak and Hasselbalch, 2011). The cytotoxic and genotoxic potential efficacy of hydroxyurea makes this molecule one of the most performing agent commonly used in chemotherapy (Spivak and Hasselbalch, 2011; Karsy et al., 2016; Liew et al., 2016). In addition, HU is usually involved in the treatment of Sickle Cell Disease (SCD) (Davies and Gilmore, 2003; Heeney and Ware, 2008; Italia et al., 2009; Flanagana et al., 2010; Candrilli et al., 2011), psoriasis (Yarbro and Leavell, 1969), Philadelphia-chromosome negative myeloproliferative syndromes (MPs) (Yarbro and Leavell, 1969), some types of solid cancers (Karsy et al., 2016), and in the therapy of HIV infection (Lori et al., 1994).

An important issue when dealing with HU is related to its harmful potential (Millicovsky et al., 1981; Woo et al., 2005) especially in prolonged exposure conditions (Elchuri et al., 2015; Broto et al., 2017), as it inhibits class I ribonucleotide reductase, leading to replication fork stalling (Quattrone et al., 2013; Liew et al., 2016). Workers involved in the manufacture of drugs, may be exposed to HU during manufacturing, transport, and distribution. In addition, as the number of patients receiving chemotherapy has considerably increased, there is a growing concern about the development of reliable, fast and accurate methods to assess the occupational exposure of workers during drug manufacturing process.

A number of analytical methods have been developed to quantify hydroxyurea in biological fluids, including spectrophotometric measurements by colorimetric techniques (Milks and Janes, 1956; Davidson and Winter, 1963; Bolton et al., 1965; Sivakumar et al., 2013; Legranda et al., 2017), electroanalytical determination (Naik et al., 2015), Nuclear Magnetic Resonance (NMR) (Main et al., 1987; Sorg et al., 2005; De Marco et al., 2011), High Performance Liquid Chromatography (HPLC) (Pujari et al., 1997; Iyamu et al., 1998; Manouilov et al., 1998), Gas Chromatography coupled to Mass Spectrometry (GC-MS) (James et al., 2006; Kettani et al., 2009; Garg et al., 2015), and Liquid chromatography—tandem mass spectrometry (LC-MS/MS) (Dalton et al., 2005; Usawanuwat et al., 2014; Marahatta et al., 2016; Hai et al., 2017). Despite the copious literature for HU detection, the assay of HU may be cumbersome due to its molecular dimension, reactivity and ability to chemical and enzymatic degradation (Iyamu et al., 1998; Marahatta and Ware, 2017).

The National Institute for Occupational and Safety Health (NIOSH) (Naumann et al., 1996) has proposed the exposure control limits (ECL) for HU not exceeding 0.01 mg/m^3^, as a consequence of the potential toxicity. Conventional chromatographic techniques (Osytek et al., 2008) usually require an accurate sample clean-up to extract HU from a filter membrane and eliminate matrix interferences. All these procedures may be critical in estimating a tiny amount of HU and may lead to sample modification (Osytek et al., 2008). To overcome these problems, spectroscopic techniques have been largely proposed to give both qualitative and quantitative information about complex samples (Zontova et al., 2016; Materazzi et al., 2017a,b). In addition, multivariate statistical analysis has already proved to be helpful in interpreting complex spectral signals (Oliveri et al., 2011; Risoluti et al., 2016a,b, 2018; Materazzi et al., 2017c).

In this work, Near Infrared Spesctroscpy is proposed as a rapid and non-destructive technique to detect and quantify HU on a glass fiber filter in order to assess a novel procedure for occupational exposure estimation. A very ultra-compact portable instrument named MicroNIR (45-mm diameter, 42-mm height and 60-g operating weight) entirely powered (5 V) and controlled via USB port of a portable computer, was used to acquire spectra; and chemometrics tools were considered to perform real-time estimation of HU. A key feature of our portable MicroNIR/Chemometrics approach is mainly related to the possibility of directly analyze samples without any pre-treatment or extraction. In addition, the method is simple and time-saving, and it can achieve the same outcomes as the conventional spectrometer.

## Materials and methods

### Materials

Hydroxyurea reference standard was purchased as powder from Sigma-Aldrich. Glass fiber filters with 2.5-cm diameter, 1-μm pore size, and 790-μm thickness (Merk Millipore) were used as membrane to collect HU. Sampling was performed by the means of a Chronos sampling device (Zambelli Srl) operated at a flow rate of 3.5 L/min for 15 min, in order to mimic occupational exposure (not exceeding 3.5 μg/filter). Reference materials were prepared in a glove-box module consisting of a cube-shaped glass box isolated from the ambient temperature and 40 μl of HU solution in deionized water at different concentrations were added to reproduce the potential amounts of HU on a filter (50, 3.5 ng, and 50 μg).

### MicroNIR/chemometrics method

Spectra were collected by a portable, ultra-compact and low-cost device MicroNIR spectrometer, developed and distributed by Viavi Solutions (JDSU Corporation, Milpitas, USA). This device operates in the spectral region 900–1,700 nm and consists of a linear variable filter (LVF) as dispersing element directly connected to a 128-pixel linear indium gallium arsenide (InGaAs) array detector and two tungsten light bulbs as radiation source.

In the MicroNIR, measuring the optimum focal point of the illumination source from the spectrometer's window to a sample is achieved by the means of a special collar. As a result, this particular geometry permits to achieve comparable outcomes as the reduction in size does not compromise the performances of the spectrometer. The instrument control was performed by the MicroNIR Pro software (JDSU Corporation, Milpitas, USA) and chemometric tools such as Principal Component Analysis (PCA) and Partial Least Square (PLS) algorithms were used as unsupervised technique and calibration models by V-JDSU Unscrambler Lite (Camo software AS, Oslo, Norway).

Spectra were collected at a nominal spectral resolution of 6.25 nm in the reflectance mode. Spectralon was used as NIR reflectance standard (blank), with a 99% diffuse reflectance, while a dark reference was obtained from a fixed place in the room. The acquisitions were performed with an integration time of 10 ms, resulting in a total measurement time of 2.5 s for each sample.

As recommended for spectroscopic data (Rinnan et al., 2009), mathematical pre-treatments were considered for chemometric evaluation such as scatter-correction methods [Standard Normal Variate transform (SNV) (Barnes et al., 1989), Multiplicative Scatter Correction (MSC) (Geladi et al., 1985), and Mean Centering (Wold and Sjöström, 1977)], Savitzky-Golay (SG) polynomial derivative filters (Savitzky-Golay, 1964) as spectral derivation techniques. Among these pre-treatments, the combination of second derivative algorithm followed by Mean Centering was selected because it provided the best outcomes in terms of Root Mean-Squared Error of Calibration (RMSEC), Root Mean-Squared Error of Prediction (RMSEP), and coefficient of determination (*R*^2^) (Miller and Miller, 2000; Mark and Workman, 2007).

Figures of merits were used to estimate model performances. In particular, Residual Predictive Deviation (RPD) was used to evaluate correction forecasting model and calculated as the standard deviation (*SD*)/RMSEP. In general, the model is considered stable when RPD ≥3 or not satisfactory when RPD < 2. In this work, the precision of the method was determined on nine different samples with concentrations regularly distributed along the linear range, using nine replicates in the same day.

Sensitivity (SEN) represents the fraction of the analytical signal responsible for an increase in the concentration of HU and was calculated as follows: SEN = 1/b, where b is the vector of regression coefficients with A latent variables. The minimum detectable concentration (MDC) is defined as the lowest concentration that can be reliably measured according to ISO 11843-2:2000 recommendations^1^.

### Experimental design

Calibration and validation models were developed using the dataset from 297 samples. The data set was divided into two groups, the calibration set (216 samples) and validation set (81 samples). In order to provide a sample selection for the calibration and validation set as representative as possible and to ensure uniformity of dataset, the X and Y distances were taken into account simultaneously, by applying the Kennard–Stone (KS) uniform sampling algorithm. The calibration set consisted of a series of reference samples including blanks (filters without HU) and fortified blanks with increasing amounts of HU (50, 3.5 ng, and 50 μg).

**Figure 1:**
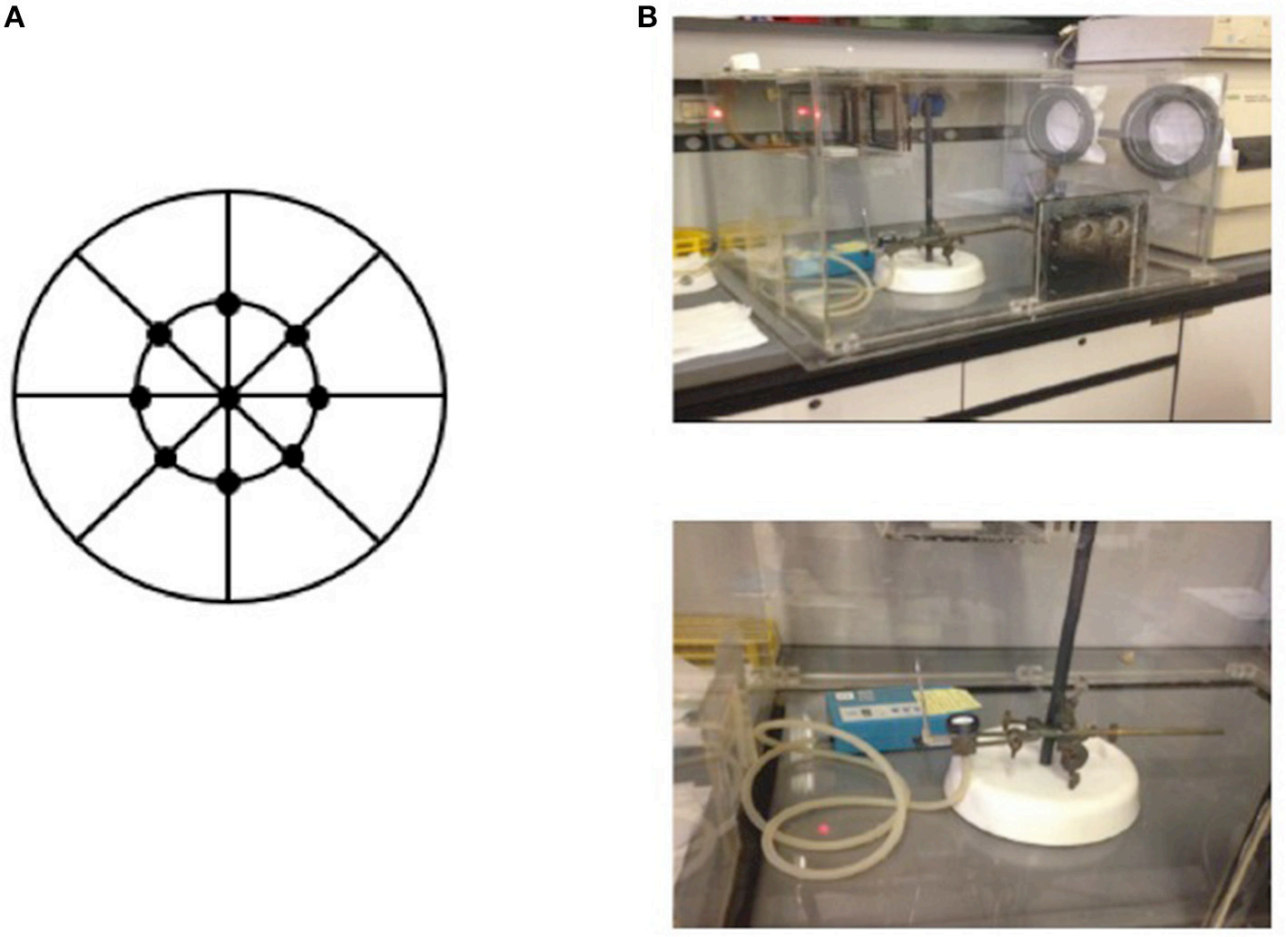
Sampling procedure of HU on a filter by acquiring nine spectra for each membrane **(A)** in a preserved glove box **(B)**.

A comprehensive sampling procedure was scheduled as follows: samples were collected in a preserved glove box and nine spectra were acquired in reflectance mode for each membrane, as shown in Figure 1. A total of nine filters were used to optimize the model of prediction for HU exposure. Six independent batches were prepared for calibration; while validation was performed on the same type of samples as the calibration set, but fully independent batches, using three series of filters.

### GC-MS method

GC-MS analysis was done on a Perkin Elmer system (Waltham, MA) using a HP-5MS (30 m × 0.25 mm × 0.25 mm) as capillary separation column. Electron impact (EI) ionization was employed at a voltage of 70 eV. The carrier gas was helium delivered at a constant flow of 1 mL/min. The oven temperature program was initially set at 150°C for 1 min, ramped to 140°C at 12°C/min and maintained for 1 min, and then ramped to 270°C at 35°C/min for 2.5 min. The temperatures for the inlet, interface, ion source and quadrupole were set at 270, 250, 230, and 150°C, respectively. Mass spectral data was collected in the scan mode from m/z 44 to 400; in the SIM mode, fragments at 277 and 292 m/z were monitored for quantification and confirmation, respectively.

## Results

To develop a novel analytical method to monitor occupational exposure to cancerogenic agents by evaluating the amount of HU on a filter, multivariate statistical analysis was performed for optimal selection of the experimental procedure. As a consequence, a number of variables were considered in order to ensure a correct and representative sampling procedure: (a) membrane type and sampling side; (b) sampling procedure to reproduce HU exposure in terms of volume to be added on a filter; (c) spectra acquisition. Preliminarily, all the acquired MicroNIR data corresponding to different experimental conditions were pre-treated and processed by a simple exploratory tool such as PCA. After that, a prediction model of HU based on Partial Least Square Regression (PLSR) was entirely developed and validated.

### Sampling procedure optimization

To make the method representative, the first investigated issue consisted of reference material preparation. Two different ways of HU deposition on a filter were investigated: (i) calibration on different filters i.e., four different filters (one blank and three fortified blanks) were considered; and (ii) calibration on a single filter i.e., only one filter was used and progressively fortified with increasing amounts of HU. In this case, spectra of blank and fortified samples were acquired prior to each deposition by the portable MicroNIR. In the first case, samples were prepared using 40 μl of aqueous solution of HU on each filter; while in the second case, a volume of 15 μl was used for each deposition.

**Figure 2:**
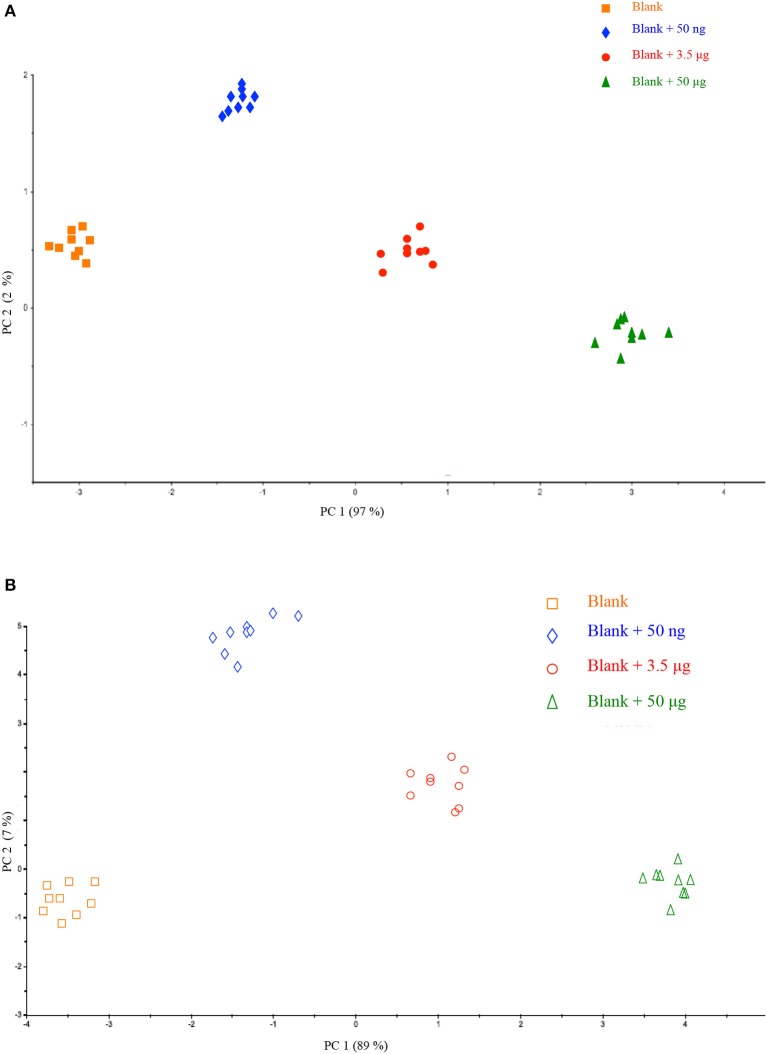
Scores plot of the Principal Component Analysis performed on the dataset related to calibration on different filters **(A)** and calibration on a single filter **(B)**.

All the acquired spectra were pre-treated and analyzed simultaneously by PCA. As displayed in Figure 2, each point represents an average of the nine respective spectra of a filter and colors were used to highlight the quantity of HU. The interpretation of the scores plot provides preliminary important information with respect to HU deposition and correlation to its different amounts on a filter.

A good correlation could be observed for samples of the same class (blank and fortified blanks) as there was no data dispersion, suggesting a correct repeatability of the method. This observation is very interesting because it is possible to clearly discriminate HU quantity on the membrane of a filter. For any deposition way, hence, the method would be suitable in practice where occupational exposure of workers may be monitored by a personal sampling system collecting a real blank (prior to HU handling) to be fortified and directly analyzed.

In addition, as shown in Figure 2, in both cases moving along PC1 (97 and 89% of explained variance) all the analyzed samples could be well grouped according to HU amount. It further confirms the ability of the approach MicroNIR/Chemometrics in monitoring occupational exposure to HU according to its amount collected on a filter.

**Figure 3:**
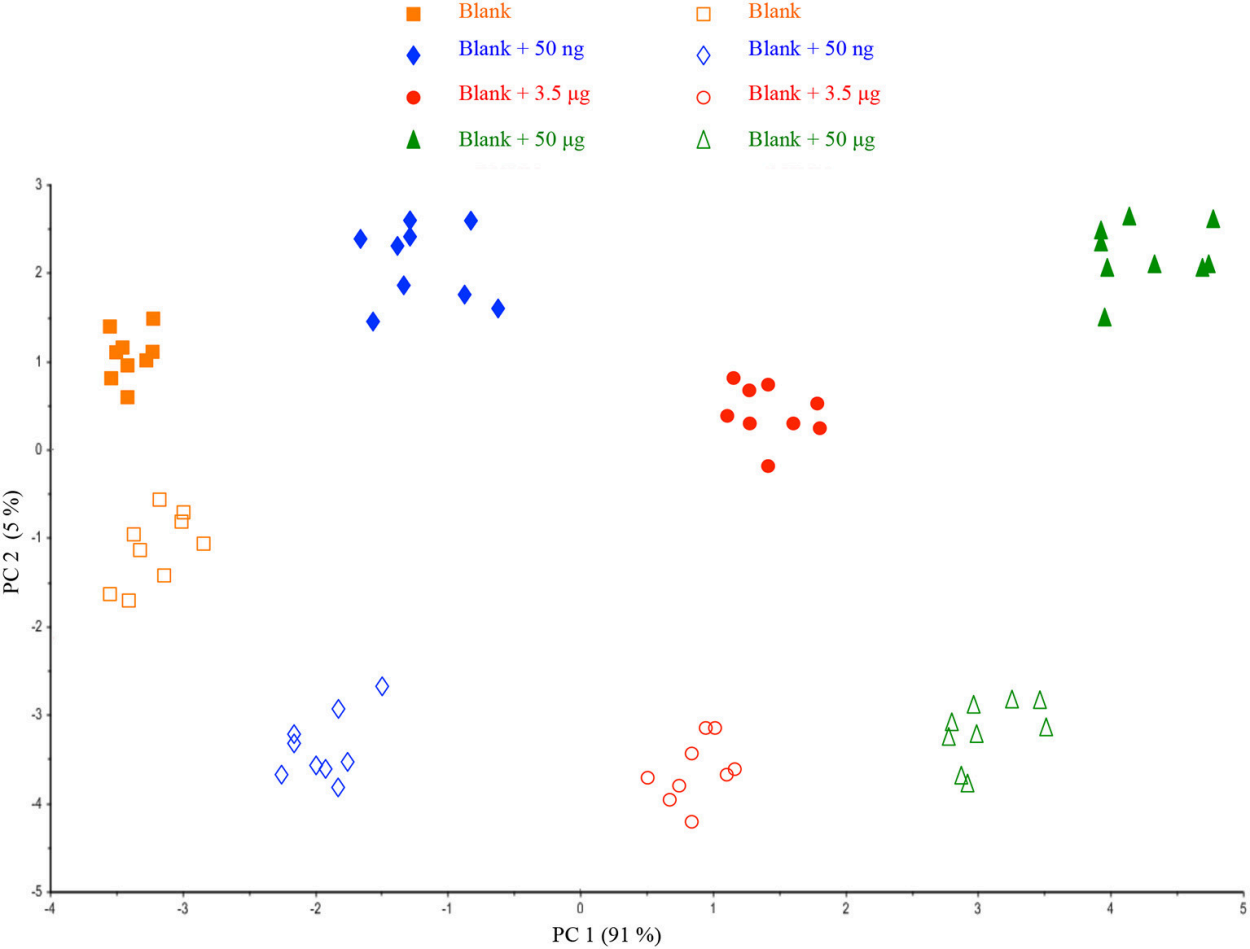
Scores plot of the Principal Component Analysis performed on the datasets from the two different ways of HU deposition on a filter.

A deeper investigation of the acquired spectra was performed by comparing the two series of samples (four- and one-filter calibration) in a single dataset. Figure 3 displays PCA data showing that the same samples can be divided into two main groups according to PC2: four- and one-filter calibration. As a result of the PCA data, the different locations of samples in the plot indicate the contribution of HU deposition way on the spectroscopic signal.

Such a result is not surprising when a reflectance acquisition mode is involved, because the surface of the filter membrane may have some influence on the spectral response as a function of the volume added. Despite the different behaviors, samples could be clearly differentiated according to PC1 (91% of explained variance) and the preliminary outcomes suggested the possibility to further investigate the repeatability of the method.

**Figure 4:**
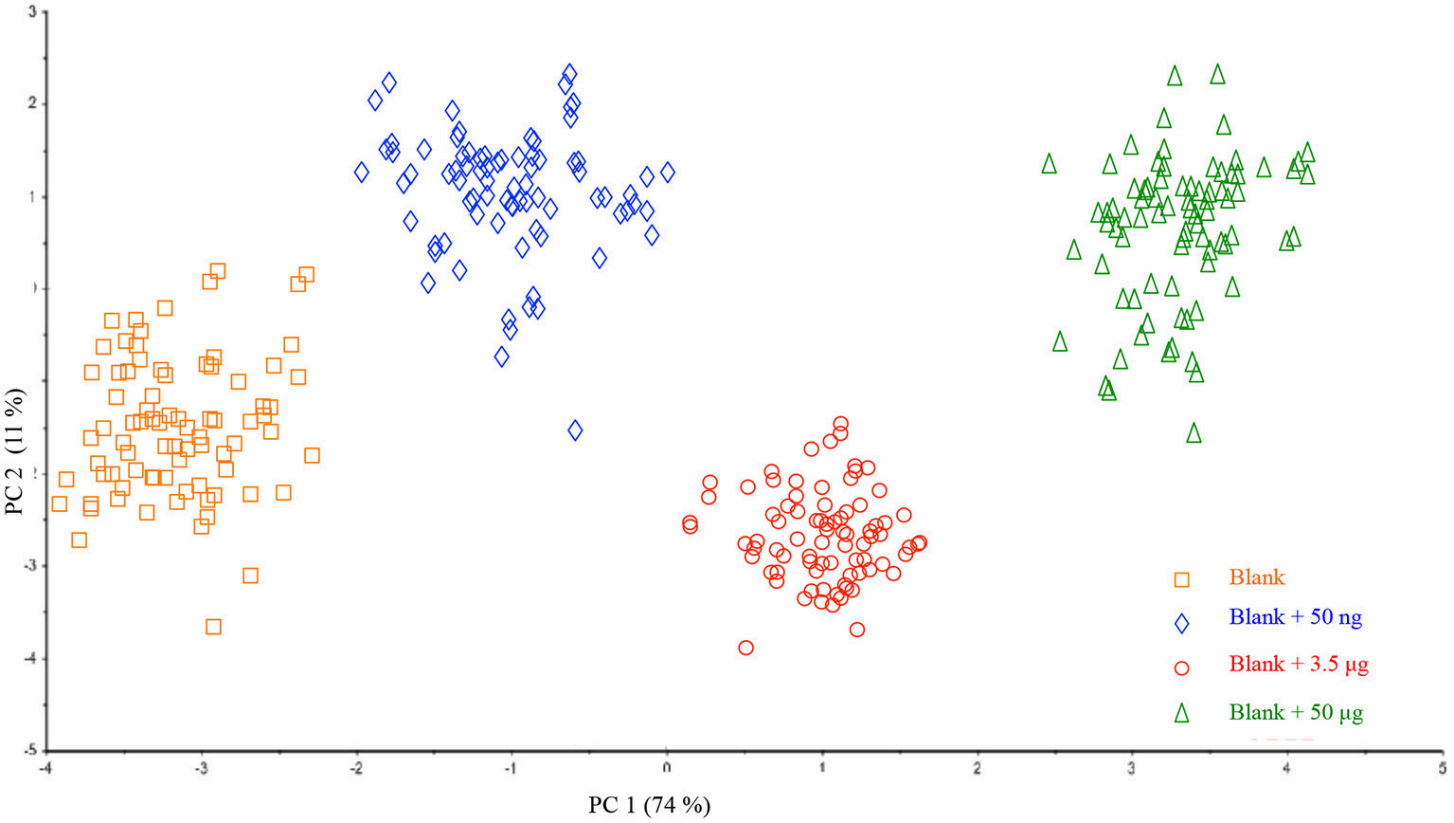
Scores plot of the Principal Component Analysis performed on the entire dataset of collected samples.

With the aim of extending this procedure to real samples, nine different filters were prepared and subsequently fortified with different amounts of HU so as to increase the number of investigated samples and evaluate whether the method would be batch-dependent. As shown in Figure 4, all the samples of the same class (displayed in different colors), could be well grouped and located in the plot according to PC1. In addition, no dispersion of data was observed thus indicating the effectiveness of the optimized HU deposition on a filter. On the basis of preliminary interesting results, a prediction model of HU on a single filter membrane was successfully validated.

### PLS model of prediction

In order to obtain the best results of calibration, the effect of a number of pre-treatments was evaluated i.e., the combination of spectral pre-treatments and wavelength range selection.

Calibration and validation sets were pre-processed using Standard Normal Variate (SNV) scaling (Barnes et al., 1989), MSC (Geladi et al., 1985), and Mean Centering (Wold and Sjöström, 1977), Savitzky-Golay (SG) polynomial derivative filters (Savitzky-Golay, 1964) and a combination of these pre-treatments.

For evaluation of model performances, comparison was made for different spectral pre-treatments to identify the most effective one in terms of prediction error using the Predicted Residual Error Sum of Squares (PRESS) to represent the sum of squares of the prediction error and the coefficient of determination (*R*^2^). Usually, the smaller the PRESS value is, the better the model's predictive ability is. *R*^2^ provides the percentage variation in y explained by x-variables and is largely used to evaluate the fitting performance. Satisfactory results (*R*^2^ and RMSEC) were obtained for the calibration of HU as shown in Table 1.

**Table 1:** Figures of merit of HU calculated with different spectral pre-treatments in calibration and prediction steps.

**Pre-treatment**	**Calibration**		**Prediction**		
	***R*^2^**	**RMSEC**	***R*^2^**	**RMSEP**	**RPD**
SNV + Mean centering	0.9985	1.98	0.9973	2.14	1.9
MSC + Mean centering	0.9889	2.02	0.9817	1.26	1.5
1st derivative + Mean centering	0.9999	0.61	0.9998	1.02	2.1
2nd derivative + Mean centering	1.0000	0.09	1.0000	0.12	5.4

**Figure 5:**
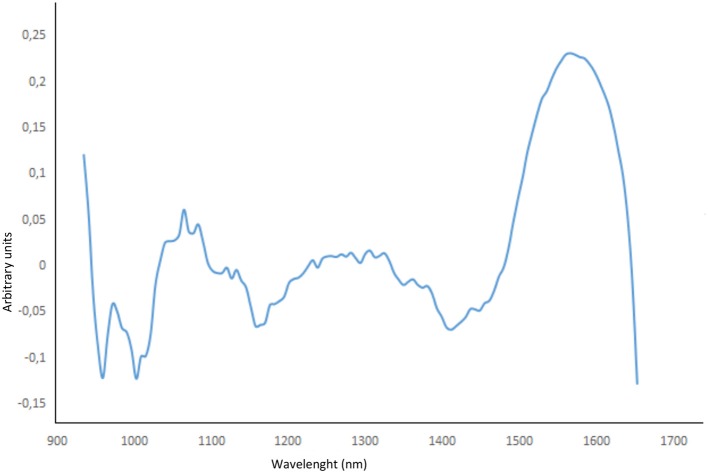
First principal component loading variation and variable spectral selection.

Good model agreement is confirmed in the validation step (*R*^2^ > 0.9817 and RMSEP < 2.14 for all the optimized models). As far as the data are concerned, the best performance can be achieved by using second derivative pre-treatment followed by mean centering (4 latent variables) as it provides the lowest RMSE and highest R^2^ values. Furthermore, the effect of the variable spectral selection within the calibration block was evaluated to improve the model's ability to predict HU. As illustrated in Figure 5, the first principal component loadings accounted for more than 87% of the total variance.

Validation results of the most performing model (second derivative pre-treatment followed by mean centering) after variable selection in the range 1,540–1,600 nm are reported in Table 2, showing that the optimized model could quantify HU on a glass fiber filter with limit of detection of 50 ng/filter. This finding points out that an adequate PLS regression model can help quantify HU directly from MicroNIR measurements without any prior sample preparation.

**Table 2:** Analytical figures of merit for PLS quantification model.

**Figures of merit**	
RMSEC	0.09
RMSEP	0.12
RPD	5.4
LV*	4
*R*^2^ Validation	1.000
Precision	1.24
Sensitivity (%w/w)^−1^	0.100
MDC** (ng)	50
Range (μg)	0.05-50
Mean ±*SD* (μg)	23.8 ± 0.65

* *Latent variables*.

** *Minimum detection concentration*.

### Evaluation of prediction ability

The validated model was consequently used to process 30 filters collected during routine HU handling. In order to evaluate the prediction ability of the model, all the samples were simultaneously analyzed by the reference method (GC-MS) and MicroNIR/Chemometrics approach. Data obtained from the MicroNIR approach (Table 3) show that the PLS model permitted to achieve the best prediction precision with RMSEP of 0.12 and RPD of 6.1, which ensured the accuracy and robustness of the model.

**Table 3:** Results of the MicroNIR approach.

**Figures of merit**	
RMSEP	0.12
RPD	6.1
Slope	0.990
Bias	0.016
Range (μg)	0.08–42.8
Mean ±*SD* (μg)	3.6 ± 0.73

In addition, the chromatographic analysis detected HU in only 19 of the 30 samples as the Limit of Detection (LOD) and Limit of Quantification (LOQ) of this method were 0.7 and 2.5 μg, respectively. When the amount of HU was chromatographically found to be lower than the LOQ of the method, LOD was used to compare with the predicted values obtained by MicroNIR/Chemometrics approach. The results showed a *R*^2^ of 0.99 and acceptable values of bias at 95% confidence (see Table 3).

MicroNIR computed values were found to be significantly lower than corresponding GC ones as the LOD of the MicroNIR method is 50 ng, meaning that the MicroNIR/Chemometrics can be a promising approach for occupational exposure monitoring at HU low levels.

## Conclusions

An ultra-compact portable device (MicroNIR) was applied to assess a novel way for HU occupational exposure monitoring.

A comprehensive sampling procedure was pointed out. Chemometric evaluation of spectra collected by a miniaturized device operated in the Near Infrared region, was optimized and entirely validated by PLS regression. The proposed method has the advantage of simplicity and avoiding sample pre-treatment, thus limiting even the analyst's HU exposure. Moreover, this approach may be considered as the optimal technology to determine cancerogenic agents or other dangerous molecules in a single-touch analysis as it is entirely portable and non-destructive. The achieved results highlight the extremely high potential of MicroNIRs to detect the HU with lower detection limits with respect to reference methods. To the best of the authors' knowledge, this approach would be the first ever proposed for the on-site detection of HU. It requires no sample preparation, is non-destructive and easy to perform (no highly-skilled personnel required), allowing a rapid evaluation of the HU occupational exposure.

## Author contributions

SM and RR conceived the study and developed the experimental design. RR performed the chemometric evaluation of data. SM and RR analyzed and interpreted data and wrote the manuscript. All authors reviewed and approved the manuscript.

### Conflict of interest statement

The authors declare that the research was conducted in the absence of any commercial or financial relationships that could be construed as a potential conflict of interest.

## Footnotes

^1^ISO 11843-2:2000. Capability of Detection, International Standards Organization. Geneva.
